# Detection of *Brucella* antibodies in domestic animals of southern Cameroon: Implications for the control of brucellosis

**DOI:** 10.1002/vms3.264

**Published:** 2020-04-03

**Authors:** Rolin M. N. Kamga, Barberine A. Silatsa, Oumarou Farikou, Jules‐Roger Kuiate, Gustave Simo

**Affiliations:** ^1^ Molecular Parasitology and Entomology Unit Department of Biochemistry Faculty of Science University of Dschang Dschang Cameroon; ^2^ Special Mission for Eradication of Tsetse flies Regional tsetse Division of Adamawa MINEPIA Ngaoundere Cameroon; ^3^ Department of Biochemistry Faculty of Science University of Dschang Dschang Cameroon

**Keywords:** *Brucella* antibodies, brucellosis, Cameroon, domestic animals

## Abstract

Brucellosis is one of the world's most widespread bacterial zoonoses caused by *Brucella.* It leads to considerable economic losses as a result of low productivity of infected animals and the long debilitating illness in humans. Despite its impact on human and animal health, little attention has been paid on *Brucella* infections in domestic animals. It is in this light that the prevalence of *Brucella* antibodies was determined in domestic animals with the overarching goal of improving our knowledge on brucellosis in southern Cameroon. During cross‐sectional studies conducted from December 2016 to August 2018 in five sites of southern Cameroon, blood samples were collected in cattle, sheep, goat, pig and dog. Plasma was obtained from each blood sample and *Brucella* antibodies were detected using the Rose Bengal test and the enzyme‐linked immunosorbent assay (ELISA). From 1873 animals that were sampled, the overall prevalence of *Brucella* antibodies using Indirect enzyme‐linked immunosorbent assay (i‐ELISA) was 6.35% (118/1873): 9.12% (78/855) in cattle; 8.04% (30/373) in sheep; 6.06% (2/33) in dog, 1.87% (3/160) in pig and 1.1% (5/452) in goat. Between animal species (*p*‐value < .0001, *x* ^2^ = 33.63) as well as sampling sites (*p*‐value = .0001, *x* ^2^ = 18.97), significant differences were observed in the prevalence of *Brucella* antibodies. Yoko and Noun localities have shown the highest prevalence of 8.6% (30/348) and 7.2% (78/1070), respectively. This prevalence was significantly higher (*p* = .03, *x* ^2^ = 1.25) in female than male cattle. Between adult (16.923%) and young cattle (7.8%), significant difference (*p* = .04, *x* ^2^ = 6.42) was observed in the prevalence of *Brucella* antibodies. This study shows that the prevalence of *Brucella* antibodies varies between animal species and localities. It also shows several domestic animals of southern Cameroon that have been in contact with *Brucella*. It enabled to identify villages where investigations on the transmission dynamic must be focused for the final goal of developing control measures for this neglected zoonotic disease.

## Graphical abstract

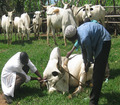

**Impacts**

- *Brucella* infections can affect cattle, pigs, sheep, goats and dogs;
- As the results of *Brucella* infections, the prevalence *Brucella* antibodies varies not only between animal species, but also between localities for which each of them has specific environmental conditions;
- Understanding the epidemiology of brucellosis for the overarching objective of designing efficient control measures requires investigating such infections on human and animal in different epidemiological settings.

## 1 INTRODUCTION

Brucellosis is a neglected anthropozoonotic disease caused by a group of bacteria of the genus *Brucella* (Aznar et al., 2015; Dean, Crump, Greter, Schelling, & Zinsstag, 2012). In livestock, *Brucella* infections cause abortion, premature birth and decreased productivity (Ayayi, Têko‐Agbo, & Koné, 2009; Havelaar et al., 2015). This infectious disease is one of the major constraints for livestock production in developing countries (Corbel, 1997; Fyumagwa, Wambura, Mellau, & Hoare, 2009). In sub‐Saharan Africa, brucellosis is considered as a serious public health problem which is responsible for tremendous economic losses estimated to be about 427 million USD per year (Mangen, Otte, Pfeiffer, & Chilonda, 2002). In this sub‐region, the prevalence of brucellosis ranges from sporadic cases to up to 41% in some affected areas (Bayemi, Webb, Nsongka, Unger, & Njakoi, 2009; Mazeri et al., 2013; Scolamacchia et al., 2010). Due to the lack of surveillance programme in many sub‐Saharan countries, many cases of *Brucella* infections are not detected (Ladbury et al., 2017). Therefore, the disease is neglected and poses an important public health threat (Ayayi et al., 2009). In most developing countries where the population growth increases steadily, the demand for livestock‐derived products such as the meat, milk and dairy products increases also in consequence (Abbasi, Abbasi, & Abbasi, 2016; Sibhatu, Krishna, & Qaim, 2015). To satisfy this demand requires improving animal production by fighting diseases that could jeopardize animal health and consequently, the quality and quantity of livestock‐derived products.

In Cameroon, previous studies have generated baseline information on cattle brucellosis in the northern part of the country (Awah‐Ndukum, Mouiche, Bayang, et al., 2018; Awah‐Ndukum, Mouiche, Kouonmo‐Ngnoyum, et al., 2018; Kelly et al., 2016; Mazeri et al., 2013; Scolamacchia et al., 2010). Despite the interesting data generated by these studies, no control strategy has been developed for brucellosis in Cameroon like in most sub‐Saharan countries. The development and implementation of control measures against brucellosis require deep investigations aiming to understand the current epidemiological situation of the disease. Although *Brucella* antibodies have been detected in cattle, no data has been published regarding *Brucella* infections in other domestic animals such as sheep, goats and pig. However, most of these animal species are susceptible to *Brucella* infections and they are also able to carry and transmit *Brucella* species that are responsible for human brucellosis.

In most sub‐Saharan countries where various domestic animal species are bred by inhabitants for which such animals constitute their main economic incomes; little attention has been paid on *Brucella* infections. Undertaking investigations on *Brucella* infections in different animal species as well as in different ecological settings could enable to generate data that will help to better understand the epidemiological situation of *Brucella* infections.

The present study was designed to improve our knowledge on *Brucella* infections in domestic animals from three agro‐ecological zones of southern Cameroon to generate data that could help to plan control strategies for brucellosis.

## 2 MATERIALS AND METHODS

### 2.1 Description of sampling sites

Domestic animals were sampled during cross‐sectional field surveys conducted in four regions (West, Central, South and South‐West) of southern Cameroon to determine the sero‐prevalence of *Brucella* antibodies in cattle, pigs and small ruminants. The first and second surveys were performed in December 2016 and June 2017 at Fontem, the third survey in August 2017 at Campo, the fourth in September 2017 at Bipindi, the fifth in November 2017 at Yoko and the sixth from April to June 2018 in the Noun Division.

**FIGURE 1:**
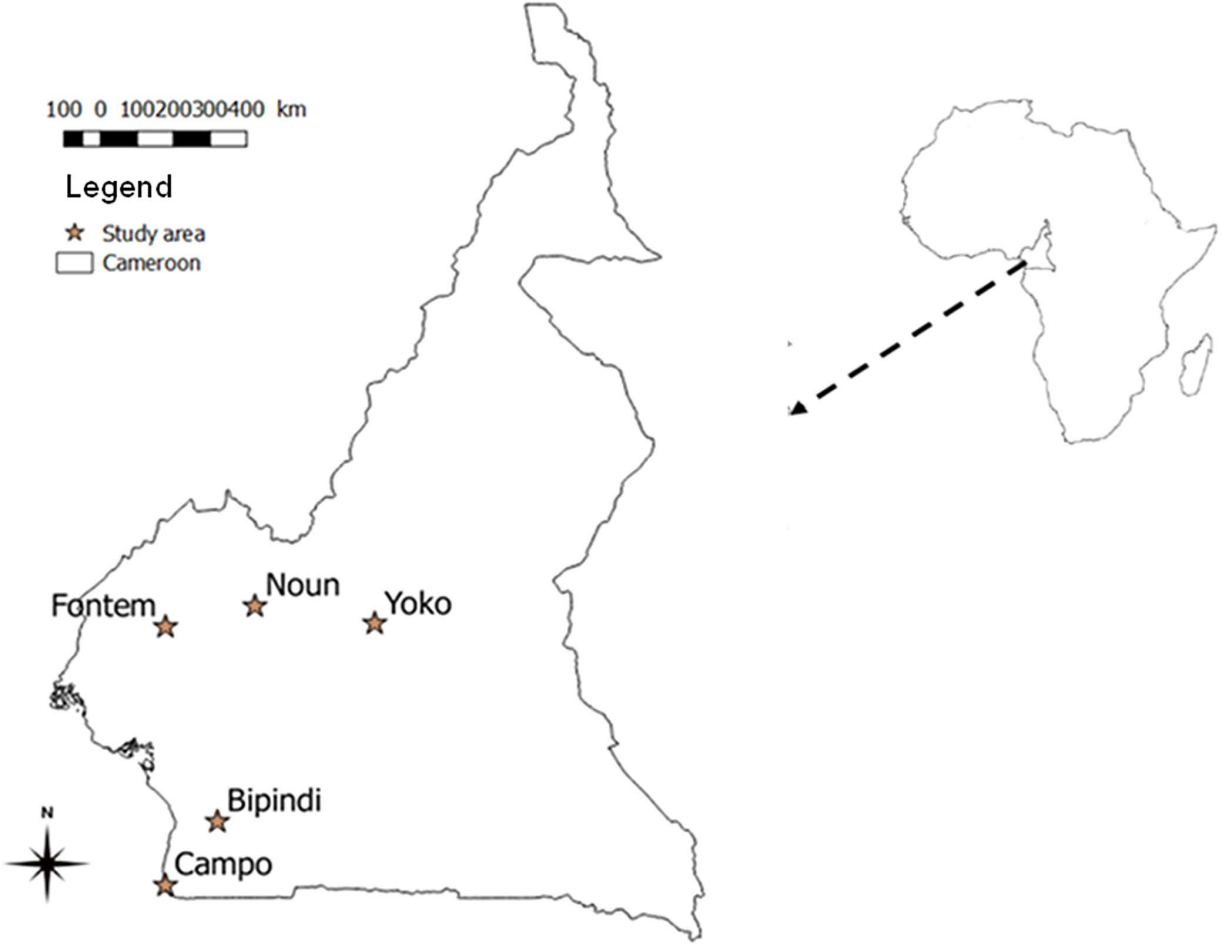
Map of Cameroon showing localities where sampling was undertaken

The Noun division **(**between 4°95′ 6°30′N and 10°30′12°E) is situated in the western highlands (Agro ecological zone III) of Cameroon (Figure 1) (Silatsa et al., 2019). Its vegetation characterized by savannah and degraded forest offers favourable agro‐climatic conditions for cattle rearing. It is therefore, the main cattle breeding area of the western region of Cameroon (Bayemi et al., 2015). Its hydrographic network is dense with streams and rivers crossing the region and the dams that, in the dry season, make it as transhumance area for farmers. The cattle reared in this locality belong to breeds such as Zebu Goudali, Zebu White Fulani and Zebu Red Fulani with few cross breeds (indigenous and exotic). Indigenous sheep and goat breeds are also kept by inhabitants. For trade or transhumance purposes, animals can move from this locality to others and vice versa and even in neighbouring countries (Bayemi et al., 2015).

Fontem (5°40′00″N, 9°55′00″E) is located in Lebialem division in south‐west region of Cameroon (Figure 1). It belongs to the agro ecological zone IV with a relief made mainly of mountains and hills (Silatsa et al., 2019). Its altitude ranges from 300 to 2,500 m above sea level. In addition to agriculture, the population also practises breeding of cattle, pigs, goats and sheep. All pigs and dogs were of local breed, originating from a mixture of different breeds.

Bipindi (3°82′00″N, 10°82′20″E) and Campo (2°82′00″N, 9°85′20″E) (Figure 1) are located in the southern region of Cameroon. They belong to agro ecological zone IV characterized by a humid forest (Silatsa et al., 2019). The main source of livelihood for people in these two localities is hunting, but inhabitants practice also small breeding of pig, sheep and goat. Cattle breeding is not developed in this area due to unfavourable climatic conditions and also animal trypanosomiasis which occurs in these areas (Nimpaye et al., 2011; Njiokou et al., 2010). The sheep and goats reared are dwarf breeds (Djallonke west‐African dwarf for sheep and Guinea goat), which are known to be trypanotolerant. Local animals live in close contact with wild animals because of their proximity with forest. Some pigs were kept in pigsties whereas other move around villages. The other domestic animal species move freely around villages.

Yoko (05°30.895′N and 012°18.830′E) is situated in the Central region of Cameroon. It is part of the agro ecological zone V (Figure 1). Considered as the large basin for cattle and small ruminants production of the centre region, its vegetation is mainly constituted by forest and savannah. Its favourable climatic conditions for animals breeding (pasture, streams and river) favour uncontrolled movements of livestock from different regions of the country as well as from neighbouring country like the Central African Republic (Motta et al., 2018; Seignobos, 2008). In this locality, sheep, goats and cattle are reared together and these animals share the same pasture and water point. The breeding system is essentially free grazing and to a lesser extent, a combination between free grazing and stall‐feeding.

### 2.2 Sample size estimation

The prevalence of *Brucella* antibodies was determined in cattle, small ruminants, pigs and dogs in Southern Cameroon. For this study, a stratified sampling strategy was applied to select herds and then, individual cattle per herd. The sample size for cattle was estimated as described by Thrusfield (2007) using the bovine brucellosis prevalence of 5.2% previously reported by Bayemi et al. (2015) in the Northwest Region of Cameroon. For this estimation, the sensitivity and specificity of the tests used were 85% and 90%, respectively as given by the manufacturer, with a precision of 0.05 and 95% confidence intervals. Only herds with a minimum of 10 cattle that were older than 6 months at the time of sampling were included. Cattle were sampled by herd and in each herd, blood samples were collected in about 20% of animals. However, more than 20% of animals of some herds were sampled due to the interests (administration of vitamins and anthelminthic to animals) and cooperation of some farmers and advices from veterinarians. From each chosen herd, the selection of cattle to be sampled was done on the basis of a systematic random sampling technique as described by Asgedom, Damena, and Duguma (2016). A total of 38 farms were enrolled in 16 villages for a sample size of 855 cattle.

For the other animal species including pigs, sheep, goats and dogs for which there is no published data on the brucellosis prevalence in Cameroon, an expected prevalence of 50% was used to estimate the sampling size. Because of the small number of goats, sheep, dogs and pigs found in each village, all of them were sampled irrespective of the number of animals presented by each household. In consequence, 452 goats, 368 sheep, 160 pigs and 33 dogs from four divisions of four regions of Cameroon were sampled.

### 2.3 Blood collection and plasma preparation

After approval from each owner, the farm characteristics and information regarding each animal including the name of the village (where each sample was collected), the geographical coordinates of each sampling site, the animal species found in farm (cattle, goat, sheep), the origin, sex, age, breed and the feeding system were recorded. Blood sample was collected from each animal by a veterinarian. Anthelmintic and vitamin were administered to these animals following advice of the veterinarian. From each animal, about 5 ml of blood was collected into EDTA‐coated tubes. This collection was performed from the jugular vein in goats, pigs, sheep and cattle and from the saphenous vein in dogs. Tubes were labelled and carefully packed to avoid crossed contamination. In the field, the blood samples were stored at 4°C in an electric cooler before being transported to the laboratory where each sample was centrifuged at 8,000 × *g* for 10 min. After this centrifugation, 500 µl of plasma was collected from each tube and then, transferred into sterile labelled micro‐tube that was subsequently stored at −20°C until use.

### 2.4 Detection of *Brucella* antibodies

*Brucella* antibodies were detected using the Rose Bengal plate test (RBPT) which is a rapid test, and the i‐ELISA test. These two tests were used to improve the accuracy or the ability to detect *Brucella* antibodies.

#### 2.4.1 Detection of *Brucella* antibodies by RBPT

The detection of *Brucella* antibodies in the plasma of domestic animals was performed with the RBPT (ID.Vet, Innovative Diagnostics) as described by Alton, Jones, Angus, and Verger (1988). Before each test, an aliquot of each plasma sample as well as the RBPT reagents were removed from the freezer and left to thaw at room temperature (22 ± 4°C) for approximately 25 min as recommended by the manufacturer. Thereafter, 30 μl of plasma and equal volume of RBPT antigen were put in each circle of the RBPT plate, and then mixed. Each plate has been allowed to rotate at room temperature for 4 min and the result was appreciated by examining the degree of agglutination. Any visible agglutination on the plate was considered positive (presence of *Brucella* antibodies in the plasma) (Nielsen & Yu, 2010). If no visible agglutination (no antibodies against *Brucella*) was observed, the test was considered negative.

#### 2.4.2 Detection of *Brucella* antibodies by i‐ELISA

The i‐ELISA test was carried out as confirmation test on all samples that were positive to RBPT to confirm RBPT results. Samples tested negative to RBPT were grouped in pool of 10 before their confirmation by i‐ELISA test. Samples from positive pools were tested individually to identify the sample that induced the positive reaction. The i‐ELISA tests were performed in polystyrene plate of 96‐wells pre‐coated with purified *Brucella* spp. antigens. Plasma samples were tested for the presence of antibodies against *Brucella* spp. *(B. arbortus, B. melitensis, B. suis* and *B. canis)* using multi species commercial i‐ELISA test kit (ID‐Screen Brucellosis Serum Indirect Multispecies, ID VET, product code BRUS‐MS‐1014, Gabrels, France). The tests were performed according to the instructions of the manufacturer (ID‐VET, 2008). Before each i‐ELISA test, reagents and plasma samples were equilibrated at room temperature (22 ± 4°C) and 100 µl of diluted buffer was added to each well. Ten microlitres of positive control and equal volume of negative control provided by the manufacturer were introduced into two different wells of the plate and 10 µl of each plasma sample was introduced in the remaining wells. Each plate was sealed and homogenized. After incubation at room temperature for 45 min, each plate was washed three times with PBS‐Tween and 100 µl of multispecies horseradish peroxidase (HRP) conjugate was added to each well. Each plate was subsequently incubated for 30 min at room temperature and washed three times to eliminate the excess of conjugate. Thereafter, 100 µl of the substrate solution (tetramethylbenzidine in substrate buffer containing H_2_O_2_) was added to each well and the plate was incubated in the dark for 15 min at room temperature. The reaction was stopped by addition of 100 μl of 1 N hydrochloric acid (HCl). The optical density in each well was measured at 450 nm using a micro plate photometer (Bio Tek ELX800 absorbance reader). For each sample tested, its results were expressed as a percentage of optical density (%OD). This percentage OD (%OD) was calculated using the following formula:

$$\%\text{OD}=100\times \left(S-N\right)/\left(P-N\right);$$

where S, N and P are ODs of the sample, the negative and positive controls, respectively. Sample with a % OD ≥ 120% where considered positive.

### 2.5 Data analysis

Statistical analyses were carried out using the Statistical Package for Social Sciences (SPSS) for Windows^®^ version 22.0 (SPSS Inc.). Kappa Cohen test was used to evaluate the concordance between RBPT and i‐ELISA tests. Chi‐squared test was used to compare the prevalence of *Brucella* antibodies between sampling areas and different animal species (pig, goat, sheep and dog) except cattle. The difference was considered significant if the *p*‐value was lower than .05.

For cattle where about 20% of animals of each herd was selected, the mixed‐effect model analysis was performed using the R package lme4. This model was performed only in cattle because they were the only animal species for which the sampling was done at the level of the herds. Pigs, goats, sheep or dogs were kept alone or by group of two or three animals in the sampled villages. For these analyses, the Bayesian Information Criterion (BIC) and log‐likelihood (logLik) for comparing models and assessing fit were considered. From these criteria; a significant fitting model was obtained when the variance of RBPT and i‐ELISA test were compared with a BIC and logLik. Results of this comparison were confirmed by analysing variance type III that was undertaken for both RBPT and i‐ELISA tests with Satterthwaite's method. The correlation was done with the analysis of variance.

## 3 RESULTS

### 3.1 Prevalence of *Brucella* antibodies in different domestic animals

A total of 1873 domestic animals containing 855 cattle, 452 goats, 373 sheep, 160 pigs and 33 dogs (Table 2) was investigated. The RBPT revealed 6.94% (130/1873) of animals with *Brucella* antibodies (Table 1). The prevalence of *Brucella* antibodies was 9.7% [95% CI: 9.6–16.4] in cattle, 9.4% [95% CI: 9.2–18.5] in sheep, 9.1% [95% CI: 2.6–18.1] in dogs, 1.87% [95% CI: 0.42–3.07] in pigs and 1.3% [95% CI: 0.48–2.90] in goats.

**TABLE 1:** Prevalence of *Brucella* antibodies according to sampling sites

Sampling sites	Number of animal tested	*Brucella* antibodies			
		Number of animals positive for RBPT (%)	95% CI	Number of animals positive for i‐ELISA (%)	95% CI
Yoko	348	35 (10.05)	9.2–18.5	30 (8.6)	7.7–16.3
Noun	1,070	85 (7.9)	6.8–16.4	78 (7.2)	6.9–14.3
Campo	54	3 (5.5)	4.8–29.0	3 (5.5)	3.1–29.2
Bipindi	118	5 (4.2)	4.2–30.7	5 (4.2)	3.6–23.6
Fontem	283	2 (0.7)	0.16–4.81	2 (0.5)	0.13–4.01
Total	1,873	130 (6.94)		118 (6.3)	
*X* ^2^		26.08		18,97	
*p* value		<.0001*		<.001*	

Abbreviations: CI, confidence interval; i‐ELISA, Indirect Enzyme‐linked immunosorbent assay; RBPT, Rose Bengal plate test.

* Significant difference in the sero‐prevalence of *Brucella* antibodies.

The i‐ELISA revealed 6.35% (118/1873) of animals with *Brucella* antibodies (Table 1). The prevalence was estimated at 9.12% [95% CI: 8.9–14.3] in cattle, 8.04% [95% CI: 7.5–16.4] in sheep, 6.06% [95% CI: 3.1–19.2] in dogs, 1.87% [95% CI: 0.36–2.59] in pigs and 1.1% [95% CI: 0.37–2.65] in goats.

The overall prevalence of *Brucella* antibodies was 6.94% (130/1873) for RBPT against 6.35% (118/1873) for i‐ELISA. Although these two tests revealed a slight difference in the prevalence of *Brucella* antibodies, the statistical analysis comparing their performance revealed a high Kappa Cohen coefficient of 0.87 with a significant *p* value (*p* < .0001); indicating a good concordance between the two tests. For its higher specificity in comparison to RBPT, the prevalence of *Brucella* antibodies in the subsequent analyses will be based on data generated by i‐ELISA test.

### 3.2 Prevalence of *Brucella* antibodies according to sampling sites

In each location of the three agro‐ecological zones where this study was carried out, at least one animal was tested positive for the presence of *Brucella* antibodies. The highest prevalence of *Brucella* antibodies of 8.6% [95% CI 7.7–16.3] was found at Yoko followed by the Noun division with a prevalence of 7.2% [95% CI 6.9–14.3]. The lowest prevalence of 0.5% [95% CI 0.13–4.01] was observed in animals sampled in villages of Fontem in Southwest region (Table 1). Between different sampling sites, a significant difference (*p *= .001, *x* ^2^ = 18.97) was observed in the prevalence of *Brucella* antibodies (Table 1).

### 3.3 Prevalence of *Brucella* antibodies according to domestic animal species

*Brucella* antibodies were detected in at least one animal of each species that were investigated in this study (Table 2). Cattle, sheep and dog were reported with the highest prevalence of *Brucella* antibodies*,* while the lowest prevalence was observed in goats (Table 2). The prevalence of *Brucella* antibodies were 8.04% [95% CI: 7.5–16.] in sheep, 6.06% [95% CI: 3.1–19.2] in dog and 1.87% [95% CI: 0.36–2.59] in pigs. Between animal species, significant differences (*p* < .05, *x* ^2^ = 33.63) were observed in the prevalence of *Brucella* antibodies (Table 2).

**TABLE 2:** Prevalence of *Bucella* antibodies according to domestic animal species

Animal species	Number of animals tested	*Brucella* infections			
		Number of animals positive for RBPT (%)	95% CI	Number of animals positive for i‐ELISA (%)	95% CI
Cattle	855	83 (9.7)	9.6–16.4	78 (9.12)	8.9–14.3
Sheep	373	35 (9.4)	9.2–18.5	30 (8.04)	7.5–16.4
Dog	33	3 (9.1)	2.6–18.1	2 (6.06)	3.1–19.2
Pig	160	3 (1.87)	0.42–3.07	3 (1.87)	0.36–2.59
Goat	452	6 (1.3)	0.48–2.90	5 (1.1)	0.37–2.65
Total	1,873	130 (6.9)		118(6.3)	
*X* ^2^		39.42		33.63	
*p* value		<.0001*		<.0001*	

Abbreviations: CI, confidence interval; i‐ELISA, Indirect Enzyme‐linked immunosorbent assay; RBPT, Rose Bengal plate test.

* Significant difference in the sero‐prevalence of *Brucella* antibodies.

### 3.4 Prevalence of *Bucella* antibodies according to intrinsic factors of animals

The prevalence of *Brucella* antibodies were 9.61% [95% CI: 7.0–13.3]) for Zebu Goudali, 11.48% [95% CI: 5.1–15.8] for Zebu Red Fulani and 7.21% [95% CI: 3.4–9.3] for Zebu White fulani (Table 3). Between cattle breeds, no significant difference (*p *= 0.78, *x* ^2^ = 0.49) was found in the prevalence of *Brucella* antibodies. However, a significant difference was observed (*p *= 0.03, *x* ^2^ = 1.25) in the prevalence of *Brucella* antibodies revealed by i‐ELISA test between female [9.94%; 95% CI: 6.5–11.1] and male [5.5%; 95% CI: 3.8–13.0] cattle. In other domestic animal species, no significant difference was observed between male and female (Table 3).

**TABLE 3:** Prevalence of *Bucella* antibodies according to intrinsic factors of animals

Animal species	Variable		Number of animals tested	*Brucella* infections				*X* ^2^	*p*‐Value
				RBPT positive (%)	95% CI	i‐ELISA positive (%)	95% CI		
Cattle	Sex	Female	694	69 (9.94)	4.5–13.1	69 (9.94)	6.5–11.1	1.25	.03*
		Male	161	14 (8.69)	4.1–11.2	9 (5.5)	3.8–13.0		
	Breed	Goudali	416	41 (9.8)	6.9–14.3	40 (9.61)	7.0–13.3	0.49	.78
		W. Fulani	291	24 (8.24)	3.1–8.3	21 (7.21)	3.4–9.3	—	—
		R. Fulani	148	18 (12.16)	6.1–14.4	17 (11.48)	5.1–15.8	—	—
	Age group	≤4 years	388	35 (9.02)	5.6–14.6	31 (7.8)	5.6–11.6	6.42	.04*
		5−8 years	402	39 (9.7)	4.6–13.5	36 (8.95)	5.6–11.5	—	—
		≥9 years	65	9 (13.84)	3.1–18.1	11 (16.92)	4.3–22.1	—	—
Sheep	Sex	Female	302	26 (8.6)	4.6–14.2	24 (7.94)	4.8–11.4	0.02	.8
		Male	71	6 (8.45)	3.0–13.4	6 (8.45)	2.2–16.4	—	—
	Age group	<1 year	29	3 (10.34)	2.0–32.2	3 (10.34)	2.1–30.2	0.3	.5
		>1 year	344	32 (9.3)	2.1–12.2	27 (7.84)	4.7–10.7	—	—
Goat	Sex	Female	401	5 (1.24)	0.0–2.7	4 (0.99)	0.0–2.5	0.3	.5
		Male	51	1 (1.96)	0.0–11.1	1 (1.96)	0.0–10.9	—	—
	Age group	<1 year	27	0 (0.00)	0.0–9.2	0 (0.00)	0.0–13.6	0.3	.5
		>1 year	425	6 (1.4)	0.0–1.9	5 (1.17)	0.0–2.7	—	—

Abbreviations: CI, confidence interval; i‐ELISA, Indirect Enzyme‐linked immunosorbent assay; RBPT, Rose Bengal plate test.

* Significant difference in the sero‐prevalence of *Brucella* antibodies

To see if the herd level may have a random effect or if there is a lack of independence in animals from the same herd, the mixed‐effect model analysis was done of data obtained from cattle. From this model, a significant fitting model was obtained when the variance of RBPT and i‐ELISA test were compared with a BIC and log‐likelihood (logLik) of‐886.5 and −154.08, a Chi‐squared test value of 68.4 and a *p*‐value of 2.2e‐16 with an intercept variance of 7.8e‐05 (CI: 0.001 ± 0.025) for the random effect on herd. These results were confirmed by variance type III analysis that revealed the statistical probability values as 2.2e‐16, F‐stat value of 2,657 and correlation of fixed effect of variance of −0.294. These results show that the herd factor has no real effect on the prevalence of *Brucella* antibodies in cattle. It is for this reason that the chi‐squared test was used above to compare the prevalence of *Brucella* antibodies between different sampling sites.

Between adult (≥9 years) [16.923%; 95% CI: 4.3%–22.1%] and young cattle (≤4 years) [7.8% 95%; CI: 5.6–13.3], a significant difference (*p* = .04, *x* ^2^ = 6.42) was found in the prevalence of *Brucella* antibodies (Table 3).

## 4 DISCUSSION

This study was designed to improve our knowledge on brucellosis by determining the prevalence of *Brucella* antibodies in domestic animals. The RBPT and i‐ELISA used here are the standard and common serological tests recommended for epidemiological study on brucellosis (Leuenberger et al., 2007). Although i‐ELISA showed higher specificity, the RBPT and i‐ELISA tests revealed a high *KAPA* value of 0.87 (*K* = 0.87); thus showing a good concordance between RBPT and i‐ELISA for the detection of *Brucella* antibodies. Our results are in line with those of Madut et al. (2018) who also observed a perfect concordance between these tests.

The significant differences (*p* < .0001, *x* ^2^ = 18.97) in the prevalence of *Brucella* antibodies between sampling sites could be explained by the variation of risk factors in various agro ecological zones. The higher prevalence of *Brucella* antibodies in animals of Yoko (agro ecological zone V) and Noun (agro ecological zone III) could be explained by the environmental conditions that are more favourable for the breeding and mixing of animals from different herds. Indeed, herd size, movement and congregation of animals for access to pastures, water or marketing figure among the well‐known risk factors of brucellosis (Berhe, Belihu, & Asfaw, 2007; Kadohira, McDermott, Shoukri, & Kyule, 1997; Megersa, Biffa, Abunna, et al., 2011; Megersa, Biffa, Niguse, et al., 2011; Mekonnen, Shewit, & Kyule, 2010; Muma, Samui, Oloya, Munyeme, & Skjerve, 2007; Sanogo et al., 2012). In the Noun division, the presence of many water streams, rivers (Noun, Mapé, Nshi, Mfû and Chiémbùh) and dams offer, especially during the dry season where water points are scarce, favourable breeding conditions that induce migration of pastoralists and their animals. This creates conditions that increase the transmission risk of *Brucella* between animals of different herds. This hypothesis is in line with observations of Khuzaima et al. (2018) reporting that when animals of different herds share temporally the same pasture zone or water point, the chance of transmission of brucellosis from infected to uninfected herds increases.

At Yoko where environmental conditions are also favourable for animal breeding, the transhumance phenomenon occurring there create favourable conditions for *Brucella* transmission due to mix up of animals from other herds or regions of Cameroon and neighbouring countries like the Central African Republic. This is in line with observations of Awah‐Ndukum, Mouiche, Bayang, et al. (2018) reporting that the mixing of large numbers of animals, the movement of animals in search of pasture, the sharing of grazing areas with wildlife, the concentration of animals around water points and the contact with other infected animals are risk factors for brucellosis spread. Sharing the same environment constitutes therefore a risk factor for *Brucella* infections and can facilitate the dissemination of brucellosis (Kaindi et al., 2012; Shimeles & Andualem, 2018).

Although the domestic animal species analysed in this study have shown susceptibilities to *Brucella* infections, significant differences (*p* < .0001, *x* ^2^ = 33.63) was observed in the prevalence of *Brucella* antibodies between different animal species. The low prevalence of *Brucella* antibodies in goat and pig could be explained by their large‐scale slaughtering for meat consumption; phenomenon that reduce the number of infected animals. Another reason will be the involvement of these animals in the intensive production systems in which they are not often in contact with infected animals or contaminated products (Kaindi et al., 2012).

It is well‐established that the dominance and overlapping nature of the C epitope of smooth brucellae (Alonso‐Urmeneta et al., 1998) makes it impossible to ascertain the infecting *Brucella* species using serological methods, irrespective of the antigen (*melitensis* or *abortus*) or host species tested (Ariza, 1999; OIE, 2013a, 2013b; Spink, 1956). Nevertheless, the presence of *Brucella* antibodies highlights contact with at least one *Brucella* species. The low prevalence (1.87%) of *Brucella* antibodies in pig is in line with previous results (Cadmus, Ijagbone, Oputa, Adesokan, & Stack, 2006; Nwanta et al., 2011; Onunkwo et al., 2011; Stafford, Tafford, Paton, & Gamble, 1992). In Nigeria for instance, the prevalence of porcine brucellosis was 0.6% (Nwanta et al., 2011; Onunkwo et al., 2011) while, in Uganda and Zambia, it was reported to be 0% (Cadmus et al., 2006; Stafford et al., 1992). Considering pig as the main host for *B. suis*, it is likely that this *Brucella* species is not highly prevalent in the sampling villages. Pig breeding could be probably not too affected by brucellosis and, like in other African countries, *B. suis* infections seem of little epidemiological importance.

Our results showing *Brucella* antibodies in dogs are the first ones reporting the possibility of dog brucellosis in Cameroon. If we consider dog as the main host of *B. canis*, our results would be in line with those of Gous et al. (2005) who reported *B. canis* in two dogs sampled in South Africa. Further investigations on *B. canis* are required to confirm this hypothesis. The probability for dogs to be infected by *Brucella* could be explained by the fact that in rural areas of most African countries where dogs are kept for many years by inhabitants for different purposes like hunting activities, these animals are in contact with wild animals. They have therefore, the possibility to become infected from wild animals that carry *Brucella* infections (Cross et al., 2018; Mick et al., 2014; Mohandoss et al., 2012).

Our results showing 9.12% of cattle with *Brucella* antibodies are in line with 8.4% reported one decade ago in the Northwest Region of Cameroon (Bayemi et al., 2009). They are lower than 31% reported in South Sudan (Madut et al., 2018), but higher than 6.8% found in Tanzania (Assenga, Matemba, Muller, Malakalinga, & Kazwala, 2015). These differences could be related to variations in the cattle management systems as previously reported elsewhere (Bayemi et al., 2015; Kaindi et al., 2012). In central Africa, most studies on brucellosis used serological tests and consequently, information related to *Brucella* species that infect cattle and small ruminants remains unknown. From data generated on bovine brucellosis in other parts of sub‐Saharan Africa, it is likely that most cattle found with *Brucella* antibodies have been in contact with *B. abortus*, the most commonly species that have been isolated and characterized in cattle from sub‐Saharan countries (Ducrotoy et al., 2017). However, it is important to point out that cattle and small ruminants share the same environment in most of our sampling sites. In such context, the transmission of different *Brucella* species can occur between cattle and small ruminants. Taking into consideration this probability, it is likely that some cattle found with *Brucella* antibodies have been in contact with *B. melitensis*. This hypothesis is in agreement with previous observations reporting the transmission of *B. melitensis* to cattle in countries where cattle and small ruminants are kept together (Benkirane, 2006; Refai, 2002). Indeed, the presence of larger herds and mixed crop‐livestock production system (cattle, goat and sheep) favours inter and intra‐species transmission of *Brucella* (Ariza et al., 2007; Mohamed et al., 2018).

The prevalence of *Brucella* antibodies in small ruminants (goats and sheep) are in line with 1.6% and 1.2% reported in Tanzania (Assenga et al., 2015) and Bangladesh (Rahman et al., 2011, 2013), but lower than the 11.4% and 5.3%, respectively reported in Sudan (Mohamed et al., 2018) and Ethiopia (Tadesse, 2016). These differences may result from the variations of risk factors such as the nomadic movements, the use of communal grazing lands and watering points for animals (Kaindi et al., 2012; Khuzaima et al., 2018). Compared to goats (1.1%), the higher prevalence of *Brucella* antibodies in sheep (8.04%) may be due to the larger herd sizes of sheep in the studied areas. Most small ruminants found with *Brucella* antibodies have been probably in contact with *B. melitensis* because, in most sub‐Saharan countries, this bacterial species have been reported as the most prevalent in these animals. However, we could not rule out the fact that *B. abortus* can be also found in small ruminants because it has been isolated several times from milk and abortion products of sheep and goat (Bertu et al., 2015; Okoh, 1980). Searching also for *B. ovis,* a non‐zoonotic species restricted to sheep, will be interesting because it has been reported to exist in several sub‐Saharan countries (Ate, Bello, Nenshi, Allam, & Rashidat, 2011; Cameron, Carles, & Lauerman, 1971; De Wet & Erasmus, 1984; Van Rensburg, Heerden, Roux, Snyders, & Heerden, 1958).

The proportion of female cattle (9.65%) with *Brucella* antibodies was significantly higher (*p* = .03, *x* ^2^ = 1.25) than male. Moreover significantly higher (*p* = .04, *x* ^2^ = 6.42) prevalence of *Brucella* antibodies was found in adult compared to young animals. These results are in agreement with those obtained in Tanzania and Pakistan (Gul, Khan, Rizvi, & Hussain, 2014; Shafee et al., 2012). These differences may result from the fact that females are kept longer in the herd for reproduction and therefore, are more exposed to infections than males (Dinka & Chala, 2009; Solorio‐Rivera, Segura‐Correa, & Sanchez‐Gil, 2007).

The main limitations of this study rely on the fact that no *Brucella* species was identified or isolated and therefore, the bacterial species circulating in different agro‐ecological zones remain unknown. However, hot spot villages were identified for subsequent investigations on brucellosis. Understanding the transmission dynamics within and between villages and between different animal species could enable to efficiently plan control operations against brucellosis. Data on this transmission could help to identify areas presenting high risk where investigations on human brucellosis could be undertaken for the overarching objective of designing efficient control programme for this neglected zoonotic disease.

## 5 CONCLUSION

The findings of this study have shown that the prevalence of *Brucella* antibodies varies between animal species and localities. Our results indicate that brucellosis can affect a variety of domestic animals from different regions of Cameroon. These results could help to identify villages where investigations on the transmission dynamic should be focused in order to improve animal health and boost peasant economy. Investigations aiming to determine the prevalence of human brucellosis and to identify *Brucella* genotypes could help to better understand the transmission dynamics of *Brucella*.

## CONFLICT OF INTEREST

The authors of this manuscript declare that they have no competing interests concerning this research.

## AUTHOR CONTRIBUTIONS

**Rolin M. N. Kamga:** contributed to conceptualization, formal analysis, methodology and writing‐original draft. **Barberine A. Silatsa:** contributed to conceptualization, investigation, methodology and writing‐original draft. **Oumarou Farikou:** contributed to data curation, investigation and methodology. **Jules‐Roger Kuiate:** contributed to conceptualization and supervision. **Gustave Simo:** contributed to conceptualization, funding acquisition, methodology, project administration, supervision and Writing‐review & editing.

## ETHICS STATEMENT

The protocol of this study was approved by the divisional delegation of the Ministry of livestock, fisheries and animals Industries of Cameroon with the reference number Ref N°015/16/L/DDEPIA.NN. The local administrative and traditional authorities of each sampling site were also informed and gave their approval. Subsequently, the review board of the molecular parasitology and entomology subunit of the Department of Biochemistry of the Faculty of Science of the University of Dschang gave its approval. Verbal consent was obtained from each owner after providing detailed explanation of the aim and objective of the study.

## Data Availability

All data generated and/or analysed are included in this article.
